# Cross-sectional and prospective association between internet addiction and risk of fatigue among Chinese college students

**DOI:** 10.1097/MD.0000000000030034

**Published:** 2022-08-19

**Authors:** Siyu Liang, Zhongyu Ren, Guang Yang

**Affiliations:** a School of Physical Education and Chinese Center of Exercise Epidemiology, Northeast Normal University, Changchun, PR China; b College of Physical Education, Southwest University, Chongqing, PR China.

**Keywords:** college students, fatigue, internet addiction

## Abstract

Severe internet addiction (IA) is associated with a higher risk of musculoskeletal pain, but whether there is a significant prospective association between IA and fatigue is unclear. This study aimed to examine the association between IA and fatigue level among Chinese college students. A cross-sectional (n = 1011) and prospective study (n = 653) was conducted to examine the association between IA and risk of fatigue. IA was measured using Young internet addiction test. Fatigue level was evaluated using the Chalder fatigue scale. Multivariate logistic regression analyses showed a cross-sectional association between IA and the risk of fatigue. The odds ratios (95% CIs) of fatigue for normal, mild, and moderate to severe groups were 1.00 (reference), 1.88 (1.20, 2.95), and 5.60 (3.33, 9.42), respectively (*P* for trend: <0.001). Similarly, multivariate logistic regression analyses also revealed a significant prospective relationship between IA and the risk of fatigue during the 1-year follow-up period. The odds ratios (95% CIs) of fatigue for normal, mild, and moderate to severe groups were 1.00 (reference), 1.56 (0.67, 3.67), and 3.29 (1.08, 10.04), respectively (*P* for trend: 0.046). Our findings indicate that IA is positively related to risk of fatigue among Chinese college students. Further interventional studies are needed to explore the causality underlying the effects of IA on fatigue.

## 1. Introduction

Fatigue is defined as a complex of physical and mental symptoms characterized by an overwhelming sense of tiredness, lack of energy, and a feeling of exhaustion, associated with impaired physical and/or cognitive functioning.^[1]^ In different populations, the prevalence of fatigue showed different trends, with a rate of 28.0% in Japanese,^[2]^ 55.2% in Korean,^[3]^ and 10.7% in Chinese adults.^[4]^ Similarly, a relatively high prevalence of fatigue was also reported in adolescents. For example, in East Asian adolescents, the prevalence of fatigue ranged from 1.6% in Korea^[5]^ to 16.5% in Japan,^[6]^ while Chinese adolescents showed substantially higher prevalence rate at 67.3%.^[7]^ Although fatigue is a suboptimal health status between health and disease,^[8]^ previous studies have indicated that exposure to long-term fatigue is associated not only with poor quality of life^[9]^ and work productivity^[10]^ but also with ischemic heart disease and stroke-induced death.^[11]^ Thus, it is essential to identify the risk factors for fatigue to develop an effective preventive strategy.

The convenience and entertainment access brought about by the widespread use of computer networks have led people to use them more frequently. Consequently, in recent years, an increasing number of individuals have been addicted to internet use. Indeed, a meta-analysis of 113 epidemiologic studies including 693,306 individuals showed that the average prevalence of internet addiction (IA) was 7.02%, and it increased over time.^[12]^ IA prevalence was higher in East Asian countries than in Western countries.^[12]^ In addition, findings from large-scale nationwide investigations and meta-analysis in East Asian countries showed high prevalence of IA, for instance, 8.1% among the Japanese^[13]^ and 12.5% among the South Korean adolescents.^[14]^ This prevalence rate of IA was close to the value in studies evaluating Chinese adolescents (11.3%).^[15]^ Moreover, our prior study found that IA was associated with a higher risk of musculoskeletal pain^[16]^ because excessive internet use, which was considered sedentary behavior, could raise systematic inflammation,^[17]^ thus subsequently increasing musculoskeletal hyperalgesia.^[18]^ Although musculoskeletal pain is a distinctive feature of fatigue, whether there is a significant association between IA and fatigue is a topic worthy of investigation. In other words, it is necessary to further explore the association between IA and the risk of fatigue.^[19–21]^ Because these results lack replication in Chinese population-based studies, we designed a cross-sectional and prospective study to examine the association between IA and fatigue level among Chinese college students.

## 2. Participants and Methods

### 2.1. Study participants

This study originated from the Chongqing Nursing Vocational College Physical Fitness and Health (CNVCPFH) study, which was carried out at Chongqing Nursing Vocational College in Chongqing, China, and aimed to examine the association between the health-related physical fitness level and the health status of college students. The Chongqing Nursing Vocational College, which includes 6 disciplinary categories (nursing science, midwifery, rehabilitation, aged people service and administration, community-based rehabilitation, and rehabilitation of traditional Chinese medicine), is the only nursing university in the municipality of Chongqing.^[22]^

In the first week of October 2018, we invited all 1094 of their freshmen population to participate in this study during their annual physical fitness measurement. All agreed to participate and provided written informed consent for the use of their data, either by themselves or with the consent of their legal guardians. All participants were asked to complete a self-designed survey questionnaire including demographic data (e.g., sex, age, father’s and mother’s educational level, parents’ marital status), lifestyle factors (e.g., breakfast frequency, smoking and drinking habits, and physical activity, sleep duration, sleep quality, and internet addiction), presence of depressive symptoms and fatigue. Eighty-three participants with missing information on lifestyle factors were excluded at baseline. During the 1-year follow-up period, we excluded 191 participants with existing fatigue at baseline and 167 participants with missing data on other variables. Finally, this study analyzed 1011 participants at baseline and 653 participants during the 1-year follow-up period. Ethics approval was obtained from the Institutional Review Board of the College of Physical Education of Southwest University.

### 2.2. Assessment of IA

Young internet addiction test (IAT) was used to assess the participants’ IA levels.^[23]^ The IAT allowed the gathering of data on the participants’ internet usage habits, their thoughts about the Internet, and the impact of internet usage on their lives. The IAT consists of 20 items, each scored on a 5-point scale (1 = “not at all” to 5 = “always”), and the scores of all the items were summed. A higher score represents more severe addiction. Three cutoff points were used to differentiate internet addiction status: normal (0–30 points), mild (31–49 points), and moderate to severe (50–100 points). The Chinese version of the IAT has been shown to have good reliability and validity.^[24]^ Cronbach α coefficient was used to evaluate internal consistency and reliability, and the results indicated that the internal reliability of the IAT was good (Cronbach α coefficient: 0.927).

### 2.3. Assessment of fatigue

Fatigue level was assessed using the 11-item version of Chalder fatigue scale (CFS).^[25]^ The CFS asked the participants to answer all 11 items with a number ranging from 0 (better than usual) to 3 (no more than usual). The scores of all items were summed to generate the total score. A higher score represents a more severe fatigue level. To define fatigue status, we dichotomize with responses “0” and “1” having scores of 0 and “2” and “3” a score of 1. A total fatigue score range from 0 to 11 and a fatigue score of ≥ 4 to define fatigue cases. The Chinese version of the CFS was shown to have good reliability and validity.^[26]^ Cronbach α coefficient was used to evaluate internal consistency and reliability, and the results indicated that the internal reliability of the CFS was good (Cronbach α coefficient: 0.866).

### 2.4. Relevant covariates

Demographic characteristics included sex and age (continuous variables) and lifestyles factors including breakfast consumption frequency (≤1 time/week, 2–5 times/week, or ≥ 6 times/week), smoking status (never, occasionally, or regularly), drinking status (never, occasionally, or regularly), and sleep duration (continuous variable) and quality (good or not). This study assessed the depressive status of participants using the Chinese version of the Self-Rating Depression Scale (SDS).^[27]^ The SDS includes 10 positive and 10 negative items, with 4 answer options (none, some of the time, most of the time, or all of the time), and each item is scored from 1 to 4. The scores of all items were summed to produce the total score, where a higher score represents a more severe depressive status. This study defined a participant with an SDS score of ≥ 50 as having depressive symptoms. Additionally, we measured the height and weight of the participants using standard protocols and computed their body mass index (BMI, continuous variable).

### 2.5. Statistical analysis

All statistical analyses were performed using IBM SPSS Statistics 24.0 (IBM SPSS Inc., Chicago, IL). Continuous variables are expressed as mean ± SD or mean (95% confidence interval [CI]), and categorical variables are expressed as percentages (95% CI). For the participants’ characteristics, analysis of variance was applied to compare the categories of IA.

Because the fatigue variable did not have a normal distribution, natural logarithm was used to normalize it for multivariate statistical analyses. Analysis of covariance (ANCOVA) was applied to analyze the association between IA categories and fatigue level after adjustment for sex and age (continuous variable) in Model 1. Model 2 was further adjusted for smoking status (never, occasionally, or regularly), drinking status (never, occasionally, or regularly), sleep quality (good or not), sleep duration (continuous variable), and BMI (continuous variable). Model 3 was further adjusted for depressive symptoms. Lastly, Model 4 was further adjusted for breakfast consumption frequency. Similarly, multivariate logistic regression analyses were used to examine the association between IA and the risk of fatigue using the above-mentioned 4 models. Statistical significance was set at *P* < .05.

## 3. Results

The participants’ characteristics according to the IA categories are presented in Table 1. Compared with participants with normal IA levels, participants with mild and moderate to severe fatigue levels had stronger drinking habits (*P* for trend: <0.001), shorter sleep duration, worse sleep quality, and more depressive symptoms (all *P* for trend: <0.001). Furthermore, the IA categories were inversely associated with breakfast consumption frequency. There were no other significant differences between the IA categories.

**Table 1:** Participants’ characteristics according to IA categories*

N = 1011	Total	Normal (n = 329)	Mild (n = 533)	Moderate to severe (n = 149)	*P*-value†
IA score	37.3 ± 12.2	25.5 ± 3.3	38.5 ± 5.2	59.1 ± 9.9	—
Female sex, %	90.0	90.0	90.1	89.9	0.998
Age, yr	18.7 ± 1.0	18.7 (18.6, 18.8)	18.6 (18.5, 18.7)	18.6 (18.4, 18.7)	0.690
BMI, kg/m^2^	21.0 ± 4.8	20.8 (20.3, 21.3)	21.1 (20.7, 21.5)	21.3 (20.5, 22.2)	0.533
Smoking status, %					
Never	92.8	92.4 (89.5, 95.3)	93.6 (91.5, 95.7)	90.6 (85.9, 95.3)	0.431
Occasionally or regularly	7.2	7.6 (4.7, 10.5)	6.4 (4.3, 8.5)	9.4 (4.7, 14.1)	
Drinking status, %					
Never	45.5	54.1 (48.8, 59.5)	42.6 (38.4, 46.8)	36.9 (29.0, 44.9)	<0.001
Occasionally or regularly	54.5	45.9 (40.5, 51.3)	57.4 (53.2, 61.6)	63.1 (55.3, 70.9)	
Sleep duration, hr	6.7 ± 1.1	6.9 (6.8, 7.0)	6.6 (6.5, 6.7)	6.3 (6.2, 6.5)	<0.001
Good sleep quality, %	75.8	82.7 (78.6, 86.8)	75.4 (71.8, 79.1)	61.7 (53.9, 69.6)	<0.001
Depressive symptoms (= >50), %	10.6	8.8 (5.6, 12.1)	7.5 (4.9, 10.1)	25.5 (20.7, 30.4)	<0.001
Breakfast consumption frequency, %					
2–5 times/week	41.3	33.1 (27.8, 38.4)	44.7 (40.5, 48.8)	47.7 (39.8, 55.5)	0.003
≥6 times/week	54.4	63.5 (58.2, 68.9)	50.8 (46.6, 55.0)	47.0 (39.0, 54.9)	0.001

BMI = body mass index, CI = confidence interval, IA = internet addiction, SD = standard deviation.

* Continuous variables are expressed as mean ± SD or mean (95% CI), and categorical variables are expressed as percentages (95% CI).

† Analysis of variance or chi-square test for continuous or categorical variables, respectively.

This study used ANCOVA to analyze whether the IA categories were positively associated with fatigue level. Table 2 shows that there was a positive and significant association between IA and fatigue level after adjustment for potential confounders at baseline. The geometric means (95% CIs) of the total fatigue level for normal, mild, and moderate to severe groups were 3.3 (2.4, 4.5), 8.4 (6.6, 10.8) and 14.8 (9.2, 24.0), respectively (*P* for trend: <0.001). Meanwhile, multivariate logistic regression analyses showed a cross-sectional association between IA and the risk of fatigue. The odds ratios (95% CIs) of fatigue for normal, mild, and moderate to severe groups were 1.00 (reference), 1.88 (1.20, 2.95), and 5.60 (3.33, 9.42), respectively (*P* for trend: <0.001) (Table 3). Similarly, multivariate logistic regressions analysis also revealed a significant prospective relationship between IA and the risk of fatigue. The odds ratios (95% CIs) of fatigue for normal, mild, and moderate to severe groups were 1.00 (reference), 1.56 (0.67, 3.67), and 3.29 (1.08, 10.04), respectively (*P* for trend: 0.046) (Table 4).

**Table 2:** Adjusted associations between IA and fatigue level at baseline.

N = 1011	Normal (n = 329)	Mild (n = 533)	Moderate to severe (n = 149)	*P* for trend*
Model 1†	3.1 (2.2, 4.2)¶	8.7 (6.8, 11.2)	15.2 (9.5, 24.3)	<0.001
Model 2‡	3.3 (2.4, 4.6)	8.6 (6.7, 11.0)	13.5 (8.4, 21.7)	<0.001
Model 3§	3.3 (2.4, 4.5)	8.4 (6.6, 10.8)	14.8 (9.2, 24.0)	<0.001
Model 4‖	3.3 (2.4, 4.5)	8.4 (6.6, 10.8)	14.8 (9.2, 24.0)	<0.001

* *P* for trend was obtained using ANCOVA.

† Model 1 adjusted for sex and age (continuous variable).

‡ Model 2 adjusted for Model 1 covariates + smoking status (never, occasionally, or regularly), drinking status (never, occasionally, or regularly), sleep quality (good or not), sleep duration (continuous variable), and BMI (continuous variable).

§ Model 3 adjusted for Model 2 covariates + depressive symptoms (categorical variable).

‖ Model 4 adjusted for Model 3 covariates + breakfast consumption frequency (≤1 time/week, 2–5 times/week, ≥6 times/week).

¶ Adjusted data are expressed as estimated geometric means (95%CI).

**Table 3:** Adjusted associations between IA and risk of fatigue at baseline.

N = 1011	Normal (n = 329)	Mild (n = 533)	Moderate to severe (n = 149)	*P* for trend*
Number of fatigue	31	93	67	—
CrudeModel	1.00 (reference)	2.03 (1.32, 3.13)¶	7.85 (4.81, 12.83)	<0.001
Model 1†	1.00 (reference)	2.03 (1.32, 3.12)	7.83 (4.79, 12.79)	<0.001
Model 2‡	1.00 (reference)	1.84 (1.18, 2.86)	6.28 (3.78, 10.45)	<0.001
Model 3§	1.00 (reference)	1.93 (1.23, 3.03)	5.73 (3.42, 9.62)	<0.001
Model 4‖	1.00 (reference)	1.88 (1.20, 2.95)	5.60 (3.33, 9.42)	<0.001

* *P* for trend was obtained using multivariate logistic regression analyses.

† Model 1 adjusted for sex and age (continuous variable).

‡ Model 2 adjusted for Model 1 covariates + smoking status (never, occasionally, or regularly), drinking status (never, occasionally, or regularly), sleep quality (good or not), sleep duration (continuous variable), and BMI (continuous variable).

§ Model 3 adjusted for Model 2 covariates + depressive symptoms (categorical variable).

‖ Model 4 adjusted for Model 3 covariates + breakfast consumption frequency (≤1 time/week, 2–5 times/week, ≥6 times/week).

¶ Adjusted data are expressed as odds ratio (95% confidence interval).

**Table 4:** Adjusted associations between IA and risk of fatigue during the 1-year follow-up period.

N = 653	Normal (n = 242)	Mild (n = 346)	Moderate to severe (n = 65)	*P* for trend*
Number of fatigue	9	18	7	—
CrudeModel	1.00 (reference)	1.42 (0.63, 3.22)¶	3.13 (1.12, 8.74)	0.045
Model 1†	1.00 (reference)	1.39 (0.61, 3.17)	3.22 (1.14, 9.05)	0.044
Model 2‡	1.00 (reference)	1.59 (0.68, 3.71)	3.48 (1.16, 10.44)	0.034
Model 3§	1.00 (reference)	1.61 (0.69, 3.75)	3.36 (1.11, 10.23)	0.040
Model 4‖	1.00 (reference)	1.56 (0.67, 3.67)	3.29 (1.08, 10.04)	0.046

† Model 1 adjusted for sex and age (continuous variable) at baseline.

‡ Model 2 adjusted for Model 1 covariates + smoking status (never, occasionally, or regularly), drinking status (never, occasionally, or regularly), sleep quality (good or not), sleep duration (continuous variable) and BMI (continuous variable).

§ Model 3 adjusted for Model 2 covariates + depressive symptoms (categorical variable) at baseline.

‖ Model 4 adjusted for Model 3 covariates + breakfast consumption frequency (≤1 time/week, 2–5 times/week, ≥6 times/week) at baseline.

¶ Adjusted data are expressed as odds ratio (95% confidence interval).

* *P* for trend values were obtained using multivariate logistic regression analyses.

When compared with participants excluded from our analysis [see Supplemental Digital Content (Appendix Table 1, http://links.lww.com/MD/H35)], the participants included in the analysis had lower BMI and lower proportion of female sex. We also analyzed change on internet addiction at baseline and follow-up, in which percentage of internet addiction had decreased trend [see Supplemental Digital Content (Appendix Table 2, http://links.lww.com/MD/H36)].

## 4. Discussion

This study examined the cross-sectional and prospective association between IA and risk of fatigue among Chinese college students. The results showed that the IA level was significantly and positively associated with the risk of fatigue after adjusting for potential confounders.

The results of this study are consistent with those of previous cross-sectional studies, which showed a positive association between internet usage duration^[28–32]^ or IA^[16]^ and musculoskeletal pain. Similarly, a significant and positive association between IA and fatigue was shown among adolescents and nurses.^[19–21]^ Compared with those studies, this study first examined the prospective association between IA and risk of fatigue in a Chinese adolescent population.

Although the exact mechanisms of fatigue development remain unclear, inflammation has been confirmed to play an important role. Several general population-based studies on younger adults found positive associations between inflammatory cytokines, such as C-reactive protein^[33–36]^ and interleukin-6,^[36]^ and fatigue level. Inflammatory cytokines can stimulate the central nervous system to produce health-compromising behaviors via neural and humoral pathways through the vagus nerve and the part of the brain with a weak blood–brain barrier, respectively. These then induce modification of neurotransmitters, neuroendocrine systems, and brain function, subsequently causing changes in behaviors (depressive mood, cognitive function decline, fatigue, etc.).^[37]^ Among these behavioral aspects, fatigue states are more sensitive to inflammatory cytokines than depressive mood and cognitive function decline.^[38]^ IA promotes inflammation also via the intake of inflammatory food. Indeed, a previous study demonstrated a linear correlation between IA level and the intake of proinflammatory food^[39]^ containing abundant proinflammatory nutrients, such as trans fatty acids and sodium.^[40]^ However, this study did not collect information regarding dietary intake. Future studies are needed to further explore the role of proinflammatory food intake in the association between IA and inflammation. However, the association between severe IA and higher fatigue levels remained significant after adjustment for breakfast consumption frequency. Since regular breakfast eaters were shown to have lower dietary inflammatory levels,^[41]^ we concluded that the significant association between severe IA and higher fatigue level was independent of breakfast consumption.

Additionally, IA had adverse effects on the fatigue level. According to its definition, fatigue can be categorized as physical and mental fatigue, with musculoskeletal fatigue and negative moods being their main manifestations, respectively.^[42]^ Our previous study that investigated the association between IA and musculoskeletal pain among Chinese young adults indicated that a severe IA status was associated with a higher risk of musculoskeletal pain.^[16]^ In China, individuals with more severe IA levels may have used the internet for longer durations. The duration of their internet usage may have accumulated by chatting with friends on WeChat or QQ software or by playing mobile or computer games. Individuals with more severe IA levels often remain in a fixed position for a long time while holding their smartphones or, more generally, show sedentary behavior. Prolonged incorrect postures can induce strain on the muscles, tendons, and disks, which raised systematic inflammation level,^[43]^ and subsequently lead to hyperalgesia in musculoskeletal system.^[44]^ Further, IA had a negative effect on mood states. Research has indicated that individuals with more severe IA have lower levels of social engagement and participation.^[45]^ Individuals who are vulnerable to IA reduce their contact and face-to-face communication with other people, which have been considered as stressors that could increase the risk of negative moods (such as anxiety, stress, and depression).^[46]^ Accordingly, we inferred that IA could have a double influence on physical and mental fatigue. We also considered several potential confounding factors, such as sex, age, BMI, smoking and drinking status, sleep duration and quality, and depressive symptoms. However, the associations between IA and fatigue after adjustment for these potential confounding factors remained significant.

## 5. Limitations

This study has several limitations. First, this observational study could not establish a causal association between IA and risk of fatigue. Second, this study only recruited regional college students from a single region, and the results may not be representative of all Chinese college students. Third, although the assessments of IA and fatigue showed good internal consistency and reliability, recall bias was inevitable owing to the subjectivity of the variables we measured. Fourth, the present study did not investigate their obligatory or elective courses status in the follow period during the semester, both of which are arranged more intensively in upper grades, and therefore could suffer from fatigue.^[47]^ Further studies are warranted to investigate these 2 kinds of course and examine whether these 2 kinds of course could confound the association between internet addiction and risk of fatigue. Finally, although we considered several potential confounding factors, we still could not completely account for the possibility that the association between IA and risk of fatigue was affected by other confounding factors such as healthy dietary habit (i.e., regular meal,^[6]^ adequate intake of eggs, fish, meat and beans; etc.^[48]^) and academic stress,^[49]^ which all correlate with risk of fatigue.

## 6. Conclusion

The results of this study indicate that IA is positively related to risk of fatigue among Chinese college students. Further interventional studies are needed to explore the causality underlying the effects of IA on fatigue.

## Author contributions

ZR, GY: conceptualization and methodology. SL: formal analysis. SL: data curation. SL: writing—original draft preparation, review, and editing. All authors have read and agreed to the published version of the manuscript.

## Acknowledgments

We would like to thank the Chongqing Nursing Vocational College students who gave their informed consent and agreed to participate in this study. We would also like to thank our staff from the Chongqing Nursing Vocational College for their dedicated work.

## Supplementary Material



## References

[1] Shen JH Barbera J Shapiro CM. Distinguishing sleepiness and fatigue: focus on definition and measurement. Sleep Med Rev. 2006;10:63–76. 16376590 10.1016/j.smrv.2005.05.004

[2] Hamaguchi M Kawahito Y Takeda N. Characteristics of chronic fatigue syndrome in a Japanese community population chronic fatigue syndrome in Japan. Clin Rheumatol. 2011;30:895–906. 21302125 10.1007/s10067-011-1702-9

[3] Yun YH Lee MK Chun HN. Fatigue in the general Korean population: application and normative data of the brief fatigue inventory. J Pain Symptom Manage. 2008;36:259–67. 18411013 10.1016/j.jpainsymman.2007.10.016

[4] Wong WS Fielding R. Prevalence of chronic fatigue among Chinese adults in Hong Kong: a population-based study. J Affect Disord. 2010;127:248–56. 20580826 10.1016/j.jad.2010.04.029

[5] Kwon M Kim H. Psychological well-being of female-headed households based on age stratification: a nationwide cross-sectional study in South Korea. Int J Environ Res Public Health. 2020;17:6445. 10.3390/ijerph17186445 PMC7559352 32899644

[6] Tanaka M Mizuno K Fukuda S. Relationships between dietary habits and the prevalence of fatigue in medical students. Nutrition. 2008;24:985–9. 18562170 10.1016/j.nut.2008.05.003

[7] Liu S Xi H-T Zhu Q-Q. The prevalence of fatigue among Chinese nursing students in post-COVID-19 era. PeerJ. 2021;9:e11154. 33954035 10.7717/peerj.11154 PMC8051357

[8] Xue X-l Wu X-y Xing J-m. Xiaopiyishen herbal extract granule improves the quality of life among people with fatigue-predominant subhealth and liver-qi stagnation and spleen-qi deficiency syndrome. Evid Based Complement Alternat Med. 2012;2012:1–9. 10.1155/2012/509705 PMC3407644 22852022

[9] Simila WA Halsteinli V Helland IB. Health-related quality of life in Norwegian adolescents living with chronic fatigue syndrome. Health Qual Life Outcomes. 2020;18:1–11. 32503553 10.1186/s12955-020-01430-z PMC7275299

[10] Sadeghniiat-Haghighi K Yazdi Z. Fatigue management in the workplace. Ind Psychiatry J. 2015;24:12–7. 26257477 10.4103/0972-6748.160915 PMC4525425

[11] Pega F Nafradi B Momen NC. Global, regional, and national burdens of ischemic heart disease and stroke attributable to exposure to long working hours for 194 countries, 2000–2016: a systematic analysis from the WHO/ILO joint estimates of the work-related burden of disease and injury. Environ Int. 2021;10659:5–106595. 10.1016/j.envint.2021.106595 PMC8204267 34011457

[12] Pan Y-C Chiu Y-C Lin Y-H. Systematic review and meta-analysis of epidemiology of internet addiction. Neurosci Biobehav Rev. 2020;118:612–22. 32853626 10.1016/j.neubiorev.2020.08.013

[13] Morioka H Itani O Osaki Y. The association between alcohol use and problematic internet use: a large-scale nationwide cross-sectional study of adolescents in Japan. J Epidemiol. 2017;27:107–11. 28142042 10.1016/j.je.2016.10.004 PMC5350616

[14] Do KY Lee KS. Relationship between problematic internet use, sleep problems, and oral health in korean adolescents: a national survey. Int J Environ Res Public Health. 2018;15:1870. 10.3390/ijerph15091870 PMC6164655 30158492

[15] Li L Xu DD Chai JX. Prevalence of internet addiction disorder in Chinese university students: a comprehensive meta-analysis of observational studies. J Behav Addict. 2018;7:610–23. 30010411 10.1556/2006.7.2018.53 PMC6426360

[16] Yang G Cao J Li Y. Association between internet addiction and the risk of musculoskeletal pain in chinese college freshmen – a cross-sectional study. Front Psychol. 2019;10 1–7. 10.3389/fpsyg.2019.01959 PMC6733990 31551859

[17] Stefansdottir R Gudmundsdottir SL. Sedentary behavior and musculoskeletal pain: a five-year longitudinal Icelandic study. Public Health. 2017;149:71–3. 28558304 10.1016/j.puhe.2017.04.019

[18] Farrell SF de Zoete RMJ Cabot PJ. Systemic inflammatory markers in neck pain: a systematic review with meta-analysis. Eur J Pain. 2020;24:1666–86. 32621397 10.1002/ejp.1630

[19] Bener A. The role of internet addiction on fatigue, sleep disturbances and poor life-style habits among adolescents. Eur Psychiatry. 2018;48:S307–S307.

[20] Bener A Yildirim E Torun P. Internet addiction, fatigue, and sleep problems among adolescent students: a large-scale study. Int J Ment Health Addict. 2019;17:959–69.

[21] Lin SC Tsai KW Chen MW. Association between fatigue and internet addiction in female hospital nurses. J Adv Nurs. 2013;69:374–83. 22515191 10.1111/j.1365-2648.2012.06016.x

[22] Ren ZY Cao JH Cheng P. Association between breakfast consumption and depressive symptoms among chinese college students: a cross-sectional and prospective cohort study. Int J Environ Res Public Health. 2020;17:1–10. 10.3390/ijerph17051571 PMC7084814 32121348

[23] Kimberly Y. Internet addiction: the emergence of a new clinical disorder. CyberPsychol Behav. 1998;1:237–44.

[24] Wu XS Zhang ZH Zhao F. Prevalence of internet addiction and its association with social support and other related factors among adolescents in China. J Adolesc. 2016;52:103–11. 27544491 10.1016/j.adolescence.2016.07.012

[25] Chalder T Berelowitz G Pawlikowska T. Development of a fatigue scale. J Psychosom Res. 1993;37:147–53. 8463991 10.1016/0022-3999(93)90081-p

[26] Jing MJ Lin WQ Wang Q. Reliability and construct validity of two versions of Chalder fatigue scale among the general population in mainland China. Int J Environ Res Public Health. 2016;13:147. 10.3390/ijerph13010147 PMC4730538 26805863

[27] Peng H Zhang Y Ji Y. Analysis of reliability and validity of Chinese version of SDS scale in women of rural area. Shanghai Med Pharm J. 2013;14:20–3.

[28] Borhany T Shahid E Siddique WA. Musculoskeletal problems in frequent computer and internet users. J Family Med Prim Care. 2018;7:337–9. 30090774 10.4103/jfmpc.jfmpc_326_17 PMC6060916

[29] Yang S-Y Chen M-D Huang Y-C. Association between smartphone use and musculoskeletal discomfort in adolescent students. J Community Health. 2017;42:423–30. 27734246 10.1007/s10900-016-0271-x

[30] Dol KS. Fatigue and pain related to internet usage among university students. J Phys Ther Sci. 2016;28:1233–7. 27190458 10.1589/jpts.28.1233 PMC4868218

[31] Hellstrom C Nilsson KW Leppert J. Effects of adolescent online gaming time and motives on depressive, musculoskeletal, and psychosomatic symptoms. Ups J Med Sci. 2015;120:263–75. 26072677 10.3109/03009734.2015.1049724 PMC4816887

[32] Ayanniyi O Ukpai B Adeniyi A. Differences in prevalence of self-reported musculoskeletal symptoms among computer and non-computer users in a Nigerian population: a cross-sectional study. BMC Musculoskelet Disord. 2010;11:177. 20691092 10.1186/1471-2474-11-177 PMC2927507

[33] Valentine RJ McAuley E Vieira VJ. Sex differences in the relationship between obesity, C-reactive protein, physical activity, depression, sleep quality and fatigue in older adults. Brain Behav Immun. 2009;23:643–8. 19133324 10.1016/j.bbi.2008.12.003

[34] Cho HJ Seeman TE Bower JE. Prospective association between C-reactive protein and fatigue in the coronary artery risk development in young adults study. Biol Psychiatry. 2009;66:871–8. 19640510 10.1016/j.biopsych.2009.06.008 PMC2763037

[35] Orre IJ Reinertsen KV Aukrust P. Higher levels of fatigue are associated with higher CRP levels in disease-free breast cancer survivors. J Psychosom Res. 2011;71:136–41. 21843747 10.1016/j.jpsychores.2011.04.003

[36] Valentine RJ Woods JA McAuley E. The associations of adiposity, physical activity and inflammation with fatigue in older adults. Brain Behav Immun. 2011;25:1482–90. 21693185 10.1016/j.bbi.2011.06.002

[37] Karshikoff B Sundelin T Lasselin J. Role of inflammation in human fatigue: relevance of multidimensional assessments and potential neuronal mechanisms. Front Immunol. 2017;8:1–12. 28163706 10.3389/fimmu.2017.00021 PMC5247454

[38] Capuron L Gumnick JF Musselman DL. Neurobehavioral effects of interferon-alpha in cancer patients: phenomenology and paroxetine responsiveness of symptom dimensions. Neuropsychopharmacology. 2002;26:643–52. 11927189 10.1016/S0893-133X(01)00407-9

[39] Kim Y Park JY Kim SB. The effects of internet addiction on the lifestyle and dietary behavior of Korean adolescents. Nutr Res Pract. 2010;4:51–7. 20198209 10.4162/nrp.2010.4.1.51 PMC2830415

[40] Allison DJ Beaudry KM Thomas AM. Changes in nutrient intake and inflammation following an anti-inflammatory diet in spinal cord injury. J Spinal Cord Med. 2019;42:768–77. 30277850 10.1080/10790268.2018.1519996 PMC6830248

[41] Haghighatdoost F Feizi A Esmaillzadeh A. Breakfast skipping alone and in interaction with inflammatory based quality of diet increases the risk of higher scores of psychological problems profile in a large sample of Iranian adults. J Nutr Sci. 2021;10:1–11. 10.1017/jns.2020.62 PMC8057508 33889393

[42] Aaronson LS Teel CS Cassmeyer V. Defining and measuring fatigue. Image J Nurs Sch. 1999;31:45–50. 10081212 10.1111/j.1547-5069.1999.tb00420.x

[43] Wilander AM Karedal M Axmon A. Inflammatory biomarkers in serum in subjects with and without work related neck/shoulder complaints. BMC Musculoskelet Disord. 2014;15:1–7. 24669872 10.1186/1471-2474-15-103 PMC3973377

[44] Afari N Mostoufi S Noonan C. C-Reactive protein and pain sensitivity: findings from female twins. Ann Behav Med. 2011;42:277–83. 21785898 10.1007/s12160-011-9297-6 PMC3184380

[45] Tullett-Prado D Stavropoulos V Mueller K. Internet gaming disorder profiles and their associations with social engagement behaviours. J Psychiatr Res. 2021;138:393–403. 33962126 10.1016/j.jpsychires.2021.04.037

[46] Esteves CS de Oliveira CR Argimon IIL. Social distancing: prevalence of depressive, anxiety, and stress symptoms among brazilian students during the COVID-19 pandemic. Front Public Health. 2021;8:1–5. 10.3389/fpubh.2020.589966 PMC7873553 33585381

[47] Yigitalp G Aydin LZ. Determination of sleep quality, fatigue and related factors in nursing students. J Nurs Midwifery Sci. 2021;8:212–8.

[48] Park SY You JS Chang KJ. Relationship among self-reported fatigue, dietary taurine intake, and dietary habits in Korean college students. Adv Exp Med Biol. 2013;776:259–65. 23392888 10.1007/978-1-4614-6093-0_24

[49] Tanaka M Fukuda S Mizuno K. Stress and coping styles are associated with severe fatigue in medical students. Behav Med. 2009;35:87–92. 19812026 10.1080/08964280903231979

