# Supplementary material for: Cross-sectional and prospective association between internet addiction and risk of fatigue among Chinese college students
Source: Medicine (Baltimore). 2022 Aug 19;101(33):e30034. doi: 10.1097/MD.0000000000030034 (PMC9387967; doi:10.1097/MD.0000000000030034)
Supplement: Supplementary file 1 [file medi-101-e30034-s001.pdf]

Appendix Table 1 baseline characteristics according to fatigue with missing values and without missing values at follow-up period.

	Followed-up (n=653)	Lost-to-follow-up (n=167)	P for trend
Female sex, %	84.4	91.3	0.009
Age, years	18.7 (18.6, 18.7)	18.6 (18.5, 18.8)	0.773
BMI, Kg/m^2	20.3 (20.0, 20.7)	23.5 (22.8, 24.2)	<0.001
Smoking status, %			
Never	93.9	91.0	
Occasionally	5.4	7.8	0.418
Regularly	0.8	1.2	
Drinking status, %			
Never	48.4	38.9	
Occasionally	51.0	59.9	0.075
Regularly	0.6	1.2	
Sleep duration, hours	6.8 (6.7, 6.8)	6.6 (6.4, 6.8)	0.103
Good sleep quality, %	81.0	77.8	0.358
Depressive symptoms (≧50), %	7.0	7.2	0.949
Breakfast consumption frequency, %			
≦1 times/week	4.0	2.4	
2–5 times/week	38.9	43.1	0.433
≥6 times/week	57.1	54.5	
