# Supplementary material for: Cross-sectional and prospective association between internet addiction and risk of fatigue among Chinese college students
Source: Medicine (Baltimore). 2022 Aug 19;101(33):e30034. doi: 10.1097/MD.0000000000030034 (PMC9387967; doi:10.1097/MD.0000000000030034)
Supplement: Supplementary file 2 [file medi-101-e30034-s002.pdf]

Appendix Table 2 change on internet addiction at baseline and follow-up

	**Baseline (n=1011)**	**Follow-up (n=653)**
**Internet addiction status**		
Normal	329 (32.5)	297 (36.2)
Mild	533 (52.7)	294 (35.9)
Moderate to severe	149 (14.7)	62 (7.6)

Values were expressed as numbers (percentage).
